# Length of Stay Analysis of COVID-19 Hospitalizations Using a Count Regression Model and Quantile Regression: A Study in Bologna, Italy

**DOI:** 10.3390/ijerph19042224

**Published:** 2022-02-16

**Authors:** Addisu Jember Zeleke, Serena Moscato, Rossella Miglio, Lorenzo Chiari

**Affiliations:** 1 Department of Electrical, Electronic, and Information Engineering Guglielmo Marconi, University of Bologna, 40126 Bologna, Italy; addisu.zeleke2@unibo.it (A.J.Z.); serena.moscato3@unibo.it (S.M.); lorenzo.chiari@unibo.it (L.C.); 2 Department of Statistical Sciences, University of Bologna, 40126 Bologna, Italy; 3 Health Sciences and Technologies Interdepartmental Center for Industrial Research (CIRI SDV), University of Bologna, 40126 Bologna, Italy

**Keywords:** count data model, length of stay, COVID-19, generalized linear model, Hurdle model, Vuong test, AIC, Rootograms, quantile regression

## Abstract

This study aimed to identify and explore the hospital admission risk factors associated with the length of stay (LoS) by applying a relatively novel statistical method for count data using predictors among COVID-19 patients in Bologna, Italy. The second goal of this study was to model the LoS of COVID patients to understand which covariates significantly influenced it and identify the potential risk factors associated with LoS in Bolognese hospitals from 1 February 2020 to 10 May 2021. The clinical settings we focused on were the Intensive Care Unit (ICU) and ordinary hospitalization, including low-intensity stays. We used Poisson, negative binomial (NB), Hurdle–Poisson, and Hurdle–NB regression models to model the LoS. The fitted models were compared using the Akaike information criterion (AIC), Vuong’s test criteria, and Rootograms. We also used quantile regression to model the effects of covariates on the quantile values of the response variable (LoS) using a Poisson distribution, and to explore a range of conditional quantile functions, thereby exposing various forms of conditional heterogeneity and controlling for unobserved individual characteristics. Based on the chosen performance criteria, Hurdle–NB provided the best fit. As an output from the model, we found significant changes in average LoS for each predictor. Compared with ordinary hospitalization and low-intensity stays, the ICU setting increased the average LoS by 1.84-fold. Being hospitalized in long-term hospitals was another contributing factor for LoS, increasing the average LoS by 1.58 compared with regular hospitals. When compared with the age group [50, 60) chosen as the reference, the average LoS decreased in the age groups [0, 10), [30, 40), and [40, 50), and increased in the oldest age group [80, 102). Compared with the second wave, which was chosen as the reference, the third wave did not significantly affect the average LoS, whereas it increased by 1.11-fold during the first wave and decreased by 0.77-fold during out-wave periods. The results of the quantile regression showed that covariates related to the ICU setting, hospitals with longer hospitalization, the first wave, and the out-waves were statistically significant for all the modeled quantiles. The results obtained from our study can help us to focus on the risk factors that lead to an increased LoS among COVID-19 patients and benchmark different models that can be adopted for these analyses.

## 1. Introduction

Coronavirus disease (COVID-19) is an infectious disease caused by the novel coronavirus SARS-CoV-2 virus [1], characterized as a pandemic in March 2020 by the World Health Organization [2]. Since the first case detected in Wuhan, dated December 2019, COVID-19 has caused 275 million cases and over 5 million deaths up to December 2021 [3]. Health systems have been significantly challenged by the spread of COVID-19, especially Italy, which was the first European country to be affected by COVID-19 [4] and which faced an overwhelming increase in COVID-19 patients who needed hospitalization at the beginning of the pandemic, risking the collapse of the system [5]. The COVID-19 pandemic highlighted the need for a management strategy to predict the demand for hospital services so that healthcare facilities can efficiently make resources available when needed.

The length of stay (LoS) is a key indicator of how efficiently hospitals are being managed and is used to assess the efficiency of hospital management and patient quality of care, and for functional evaluations. A shorter stay means that more beds are available for more patients and reduces hospital resource consumption; thus, it corresponds to a decrease in health-related expenditure [6]. Reducing LoS has been linked to lower risks of opportunistic infections and medication side effects [7], and improved treatment outcomes and lower mortality rates. In addition, a shorter hospital stay reduces the burden of medical fees and increases bed turnover, and thus increases hospitals’ profit margins [7] while lowering the overall social costs [8,9,10]. Researchers must determine which characteristics are associated with longer or shorter hospital stays in patients [11]. 

A recent systematic review and meta-analysis [12] research compared the LoS of COVID-19 patients in China versus the rest of the world (Europe, the U.S., and the UK) using 52 studies. By combining information from different studies, a substantial difference in LoS between China (longer LoS) and other locations (shorter LoS) was found. There is also little evidence concerning the impact of the study period, age, and disease severity on LoS. Another retrospective study in Vietnam found that age was significantly associated with a long hospital stay in COVID-19 patients [13]; other studies in China found that the average age of patients with a prolonged LoS was higher than that of patients with an average LoS [14]. A better understanding of the COVID-19 pandemic’s spillover effects between waves might help healthcare professionals better support at-risk persons during the pandemic. Study results have indicated that there is a minor difference in overall LoS before and after the years of the COVID-19 pandemic according to a territory-wide retrospective cohort study conducted in Hong Kong on 28-day in-hospital mortality and LoS. In 2020, patients triaged as critical in the emergency department (ED) had a 2.71-day shorter LoS than those in 2019. LoS declined significantly for patients who died in 2020, when their hospital stay was 2.31 days shorter on average than in 2019 [15].

Through univariate and multivariate logistic regressions models, a retrospective analysis of patients hospitalized with COVID-19 from a single hospital in Hefei, China, was carried out to explore the risk variables associated with a prolonged hospital LoS. The findings revealed that patients with a prolonged LoS had a higher average age than those with a median LoS [13]. A time-to-event analysis implementing a Cox proportional hazard model was applied to identify the factors associated with the LoS. Factors linked to a longer hospital stay should be considered when arranging bed strength on a contingency basis, and the estimated LoS of patients was required in order to model bed occupancy and make contingency plans. Artificial Intelligence (AI) researchers developed algorithms in order to model bed occupancy and make contingency plans [16]. Another study in South Korea used multivariate negative binomial and gamma regression models to determine the influencing factors of LoS [17]. The results showed that the average LoS in hospitals for COVID-19 patients was 5.5 days, and the average LoS also tended to be higher for older patients, except for the group aged 65 years or older, who had a shorter hospital stay than the others. A study in the UK used an Accelerated Failure Time (AFT) survival model, a truncation corrected (TC) method, both with underlying Weibull distributions, and a multi-state (MS) survival model to analyze LoS data [18]. The results showed that all methods produced similar estimates of LoS for the overall hospital stay, considering ordinary hospitalization only: 8.4, 9.1, and 8.0 days for AFT, TC, and MS, respectively. This study also tried to mention the complexities and partiality of many data sources, as well as the continuously developing nature of the COVID-19 pandemic that suggests approaches should be based on a variety of analytical methodologies across multiple datasets. From a management point of view, research in Dubai used AI-based modeling by using Decision Tree prediction models to forecast the LoS and risk of mortality accurately. In principle, these smart models might equip front-line clinicians with the tools they need to improve management techniques and save lives [19].

Researchers have been focusing on modeling count data due to observed problems with the commonly used analytic methods. For example, ordinary least squares (OLS) regression is often inappropriate because the count data violate the underlying assumptions of OLS regression (i.e., the normality of the residuals), resulting inaccurate coefficient estimates and standard errors, and misleading *p*-values and consequent confidence intervals [20,21]. 

Only recently, several studies have examined the effective management of LoS, and several statistical models have been proposed to analyze the data with count-type outcomes (such as LoS in the hospital, number of antenatal care visits, number of purchases made, and many more) with different settings and predictors [22,23]. There are, however, different characteristics in different count data; therefore, certain count data models cannot be used with them. Often, the first model used to analyze count outcomes is the Poisson model [10,20] but, in practice, these two assumptions (i.e., the variance being equal to mean, and independent events) [10,20] are typically violated. Overdispersion is generally always the result of any assumptions being violated [20,24,25], and alternate strategies for modeling count data, such as negative binomial regression [20,24,25], should be considered. Aside from overdispersion, many empirical counts contain a large number of zero observations, which can be modeled using Hurdle models [26]. A Hurdle model combines a binary count outcome (LoS = 0 vs. LoS > 0) with a truncated model for results above the hurdle (LoS > 0). Excess zeros are modeled using the Hurdle model. In some cases, these zeros cannot be ignored, since they are critical and meaningful. To assess the fitted count regression model, researchers have used a graphical approach called Rootograms [27,28].

Another statistical method used to characterize the effects of covariates on a given phenomenon is to study the quantiles’ distribution instead of modeling the mean. Quantile regression models estimate the relationship between the qth quantile of the response *y* and the covariate *x* [29]. Quantile regression models at multiple percentiles can help compare the changes associated with covariates across the distribution of the phenomenon under study [30]. Machado et al. [31] extended quantile regression models for count data. This approach has already been applied in a study [32] to evaluate the LoS of diabetes patients. 

This study aimed to identify and explore the hospital admission risk factors associated with LoS in COVID-19 patients in Bologna, Italy, by applying a relatively novel statistical method (i.e., count regression and quantile regression models) using the currently available predictors. The second goal of this study was to model the LoS of COVID patients in Bologna hospitals from 1 February to 10 May 2021 in order to determine which covariates had a significant impact on it and to discover the potential risk factors linked with LoS. 

## 2. Materials and Methods

### 2.1. Population

This study obtained data with permission from the local health authority (AUSL) of Bologna. Data were retrieved from 1 February 2020 to 10 May 2021. Our dataset referred to COVID-19 patients both in the ICU (intensive plus sub-intensive) and ordinary settings in 7 Bolognese hospitals. We also categorized the hospital stays as “regular” for 5 hospitals, and “long-term” for 2 hospitals. This distinction is important because hospitals providing extended hospitalization have mainly rehabilitation purposes.

### 2.2. Database Preprocessing

Individual patients were considered on the basis of their unique ID, and we merged the repeated IDs (patients who went to the hospital more than one time) via the following procedure:

- Detect repeated ID;
- Order rows referring to the same ID chronologically;
- Check if two consecutive rows differ for >3 days
- If they differed for >3 days, the two rows were treated as two different stays. The second stay was removed from further analysis;
- If they did not differ for >3 days, merge multiple rows by using:
- Hospital: prevalent hospital
- Setting: add a variable with two levels: “low-intensity” if the patient was only hospitalized in the low-intensity setting and “ICU” if the patient was in the intensive care unit at least one time.

We categorized the hospital stay information as regular hospitalization and long-term hospitalization based on the prevalent hospital. 

### 2.3. Outcome and Covariates

The study’s main outcome was the LoS (a non-negative integer), defined as the number of days between inpatient admission and hospital exit or discharge, and it was the target outcome variable for which this study aimed to identify a proper count regression model. The explanatory variables were:

Clinical setting: ICU setting (intensive care + sub-intensive) of COVID-19 patients, and ordinary and low-intensity COVID-19 stays. 

Age: An integer representing patient age in years, grouped in 10-year categories: [0, 10), [10, 20), [20, 30), [30, 40), [40, 50), [50, 60), [60, 70), [70, 80), and [80, 102). 

Waves: We defined waves based on an empirical approach. 

**Figure 1:**
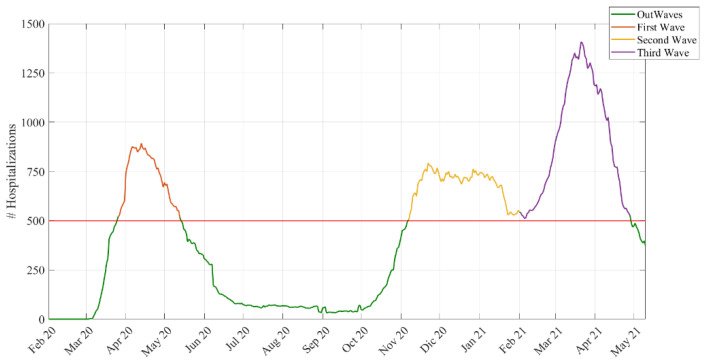
Number of hospitalizations over the study period and the empirical definitions of the waves.

We assumed that a wave started with more than 500 total hospitalizations and ended when the hospitalizations dropped below 500 (see Figure 1). Using this cut-off, we chose a day in the stationary phase between the second and the third wave located in the middle of this period. We chose a middle point to divide the second from the third wave, since hospitalization in that period never dropped to less than 500. Using this criterion, these were the dates and durations of the waves:

First wave → 26 March 2020–13 May 2020 (48 days)

Second wave → 7 November 2020–1 February 2021 (86 days)

Third wave → 2 February 2021–28 April 2021 (85 days)

Out-waves → other periods.

Hospital stay: A factor containing hospital name and categorized as long-term or regular hospitalization. 

### 2.4. Ethical Considerations

Ethical approval for the study was obtained from the University of Bologna’s Ethical Committee (approval number 283066, 5 October 2021).

### 2.5. Statistical Analysis

#### 2.5.1. Poisson and Hurdle Models

The generalized linear model (GLM) is a framework for regression models with a wide range of outcome variable types [33]. A *p*-value of ≤ 0.05 was deemed to be significant. The outcome variable, LoS, is a count type, and Poisson regression is the most common type used to analyze this kind of data. However, in practice, the Poisson assumptions are usually violated (there is overdispersion), and it was revealed that the Poisson model did not fit the data, so alternative models such as the negative binomial model were considered [20,24,25]. Both the Poisson and NB models were implemented in R Studio with R version 4.0.4. [34] by the glm function in the stats package and the glm.nb function in the MASS package.

In addition to overdispersion, many count data have more zero observations. As a result, if the proportion of zeros in the response variable is not high, the Hurdle model would be a preferable option [26].

This is a two-stage model:

1. Zero vs. non-zero (logistic regression);
2. Regression of counts > 0 (zero-truncated Poisson or NB).

It combines a count data model $f_{count}\left(y;x,\beta \right)\;$(left-truncated at y=1 and a zero Hurdle model $f_{zero}\left(y;z,\gamma \right)$ (right-censored at y=1):

$$f_{hurdle}\left(y;z,\beta ,\gamma \right)=\{\begin{array}{ll} f_{zero}\left(0;z,\gamma \right) & if\;y=0\\ \left(1-f_{zero}\left(0;z,\gamma \right)\right)f_{count}\left(y;x,\beta \right)/\left(1-f_{count}\left(0;x,\beta \right)\right) & if\;y>0 \end{array}$$

where y is the value of the dependent variable, z is a vector denoting the predictor variable in the zero Hurdle model, x represents a vector denoting the predictor variable in the count data model, γ is a vector of the coefficients related to z, and β denotes a vector of coefficients related to x. $f_{zero}$ is a probability density function of y=0, typically modeled with binary logistic regression, where all counts greater than 0 are given a value of 1 and 0 otherwise [26]. In R Studio (R version 4.0.4), Hurdle count data models are fitted with the hurdle function of the pscl package [35].

#### 2.5.2. Model Selection and Comparison

We compared the performance of various count models to determine which one was the best based on the following criteria:

1. Akaike Information Criterion (AIC):

For non-nested models, the standard approach is to use information criteria such as the AIC [36,37] and the Bayesian Information Criteria (BIC) [37]. The AIC estimates the quality of each model based on the formula: AIC=2k−2logL(θ), where L(θ) is the maximized likelihood function for the estimated model and k is the number of parameters in the model. Given a set of candidate models for the data, the preferred model has the minimum AIC value [36].

2. Vuong test:

The purpose of the Vuong test [38] is to compare two models (that are not nested) fitted to the same data by maximum likelihood, and it is based on a comparison of the predicted probabilities of two models that are not nested. Specifically, it tests the null hypothesis that the two models fit the data equally well. A large positive test statistic provides evidence of the superiority of Model 1 over Model 2, while a large negative test statistic is evidence of the superiority of Model 2 over Model 1, under the null hypothesis that the models are indistinguishable.

Let $p_{1}$ be the predicted probabilities from Model 1, evaluated conditionally on the estimated MLEs. Let $p_{2}$ be the corresponding probabilities from Model 2. The Vuong statistic is:

$$\mathrm{Vuong}={\left(s_{m}\right)}^{-1}\sqrt{N}\;m$$

where $m=\log\left(p_{1}\right)-\log\left(p_{2}\right)$, $s_{m}$ is the sample standard deviation of m, and *N* is the sample size [9]. We compared all the count models in R Studio with R version 4.0.4. using the pscl package [35]. 

3. Rootograms:

Rootograms is a graphical approach used to assess the fit of a count regression model. It is useful for diagnosing and treating issues such as overdispersion and/or excess zeros in count data models [27,28]. We visualized the plot in R Studio with R version 4.0.4. using the countreg package [27,28].

#### 2.5.3. Quantile Regression Model

A quantile regression model can be used to explore the relationships between the quantiles of the response distribution and the available covariates. By comparing such quantiles, we can obtain a more complete picture of the conditional distribution than we can with regression models that consider the mean. In addition, quantile regression allows researchers to explore a range of conditional quantile functions, thereby exposing various forms of conditional heterogeneity and controlling for unobserved individual characteristics.

With a Poisson distribution, quantile regression models were used to model the effects of covariates on the quantile values of the response variable, LoS. A linear quantile regression model was fitted to the log-transformed response. The modeled quantiles were 0.25, 0.5, 0.75, 0.8, 0.9, and 0.95. We used the quantreg package in R [39]. 

## 3. Results

### 3.1. Descriptive Statistics

Descriptive statistics were used to summarize the baseline characteristics of the study population. In our setting, a total of 13,203 COVID-19 patients were included in the study. The median length of stay during the study period was 6 days. Table 1 summarizes the descriptive statistics and the characteristics of the variables considered in the study. 

**Figure 2:**
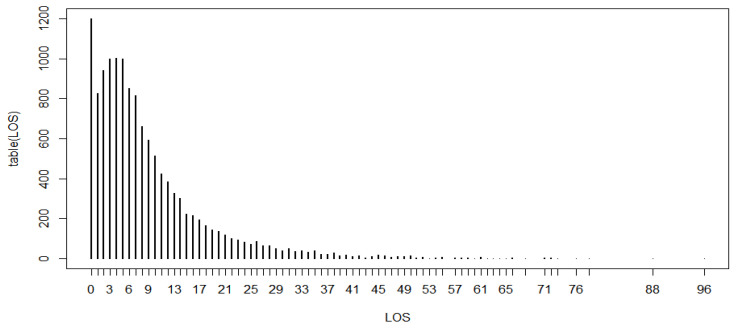
Frequency distribution of LoS.

Figure 2 shows the frequency distribution of LoS, which was right-skewed.

**Figure 3:**
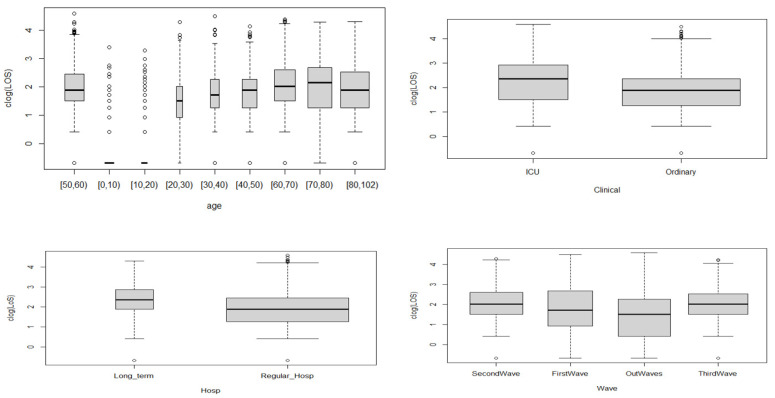
LoS plotted against the predictors used. All plots show the variation in LoS when the predictors were changed.

**Figure 4:**
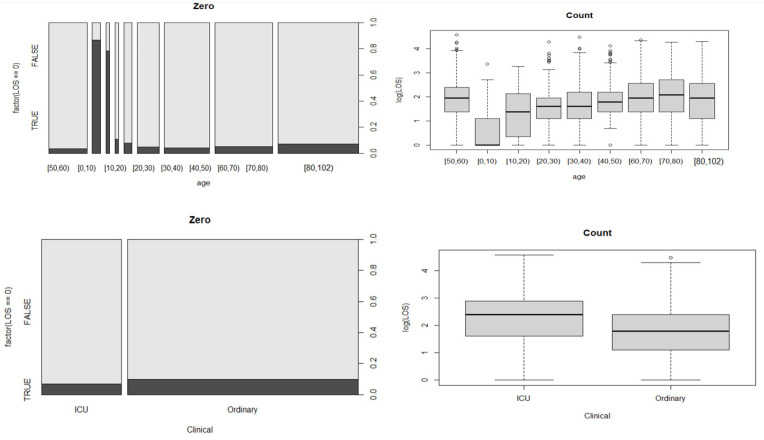

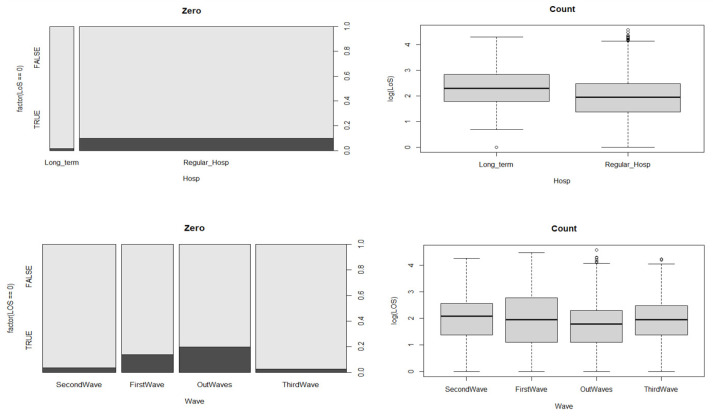
Plots of (LoS = 0 vs. LoS > 0) for each predictor.

Figure 3 shows the variation in LoS for each predictor. In Figure 4, the plots of LoS = = 0 (true) vs. LoS > 0 (false) are displayed for each predictor. True values (in black) indicate that zeros are infrequent except for the age groups [0, 10) and [10, 20). False values (in white) indicate that LoS values greater than zero are more frequent, and these are shown in the count boxplot for each predictor.

### 3.2. Count Regression Models

#### 3.2.1. Results of the Poisson and Hurdle Models 

Table 2 shows the values of the parameter estimates (and the standard errors in brackets) of the Poisson and negative binomial(NB) models. All predictors were significant for the Poisson model, but there was overdispersion and poor data fit. In contrast, the NB model fitted the data well compared with the Poisson model, and all predictors were significant, except for the third wave category among the wave predictors (see Table 2). 

Table 3 reports the coefficients of the fitted models. The second column of the table reports the count process of the Hurdle–Poisson model; the third column shows the count process of the Hurdle–NB model. In Section A, only the truncated process, i.e., positive counts, has been fitted. In Section B, zero counts were fitted through a binary logit model.

#### 3.2.2. Model Selection and Comparison

A structured model selection and comparison process is crucial to avoid misleading results and interpretations. The final interpretations were based on the selected model.

##### Vuong Test

The comparison between the NB and Poisson models had a Vuong test statistic of 39.50 with a *p*-value < 0.0001, indicating that the NB model provided a better fit. Similar results were obtained for the other models. From Table 4, we can conclude that the preferable model is the Hurdle–NB model because it has the highest value of the Vuong test statistics and a significant *p*-value (see Table 4).

##### AIC/BIC

The Poisson regression model had the largest AIC value but the worst fit to the data. Hurdle–Poisson had the second largest AIC, whereas Hurdle–NB had the smallest AIC value and hence was the best choice for this dataset (see Table 5).

##### Rootograms

**Figure 5:**
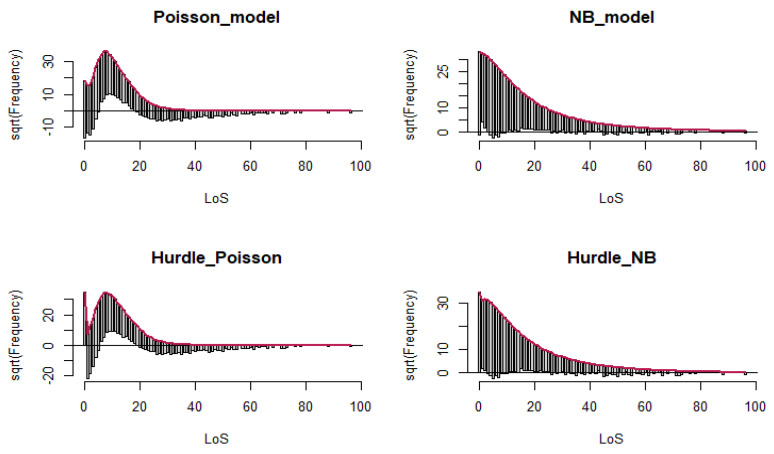
Rootogram plots.

The hanging Rootograms for the Hurdle–NB showed a much better fit with the data than other models. The second best was NB, but some of the zero counts were underfitted (under the line) and the LoS counts were also overfitted (over the line), as in [25], the line at 0 allows us to easily visualize where the model is over- or underfitted. At 0, it fits perfectly by design. The line above indicates that the variability of our observed data is much greater than what a Poisson model would predict. The red line is the theoretical Poisson fit. “Hanging” from each point on the red line is a bar, the height of which represents the difference between the expected and observed counts (see Figure 5).

The regression exponential coefficients (exp(coef)), i.e., the incidence rate ratios (IRR), give information about the increase in the expected LoS for a unit of increase in a given predictor. Hence, an IRR greater (smaller) than the one obtained for the predictors suggests longer (shorter) expected LoSs for the selected models.

Interpretation of NB_IRR (see Table 6): The baseline or the average intercept LoS count is 7 days. The other exponentiated coefficients are interpreted multiplicatively. On average, the ICU setting increased LoS by 1.77-fold compared with ordinary hospitalization and low-intensity stays. Long-term hospitals increased the average LoS by 1.62 compared with regular-stay hospitals. Average LoS decreased by 0.06-, 0.17-, 0.84-, 0.86-, and 0.87-fold in the age groups of [0, 10), [10, 20), [20, 30), [30, 40), and [40, 50), respectively, compared with the intermediate age group of [50, 60), and increased by 1.15-, 1.12-, and 1.11-fold in the age groups of [60, 70), [70, 80), and [80, 102), respectively. The third wave did not have a significant effect on the average LoS, which increased by 1.11-fold during the first wave and decreased by 0.77-fold during the out-wave compared with the second wave (chosen as the reference level).

Interpretation (Hurdle–NB, zero-Hurdle model) (see Table 6): The baseline or the intercept odds having a positive count vs. zero were 47.57. The odds of having a positive count vs. zero in the ICU setting did not significantly affect ordinary hospitalization and low-intensity stays. The odds of having a positive count vs. zero increased by 3.61-fold for every unit of increase in long-term stays in hospital compared with regular stays in hospital. The odds of having a positive count vs. zero decreased by 0.34- and 0.39-fold during the first wave and out-wave compared with the second wave, whereas the third wave did not have a significant effect. The odds of having a positive count vs. zero decreased by 0.01-, 0.02-, 0.40-, 0.49-, 0.64-, and 0.44-fold in the age groups of [0, 10), [10, 20), [20, 30), [30, 40), [70, 80), and [80, 102), respectively, whereas the age groups of [40, 50), [60, 70) did not have a significant effect.

**Figure 6:**
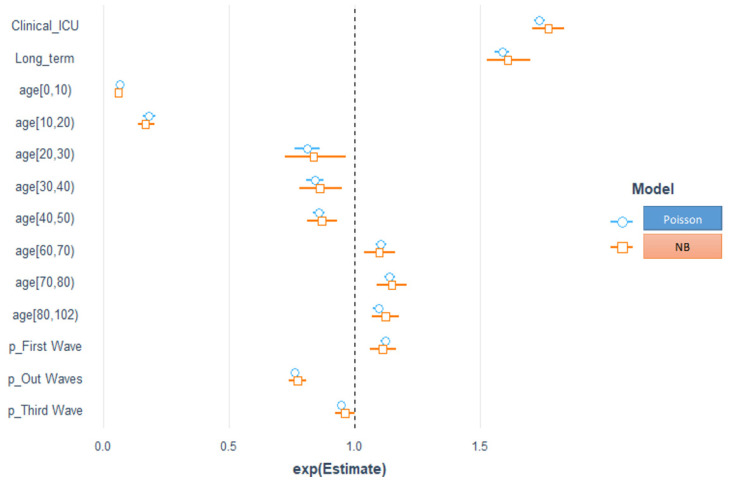

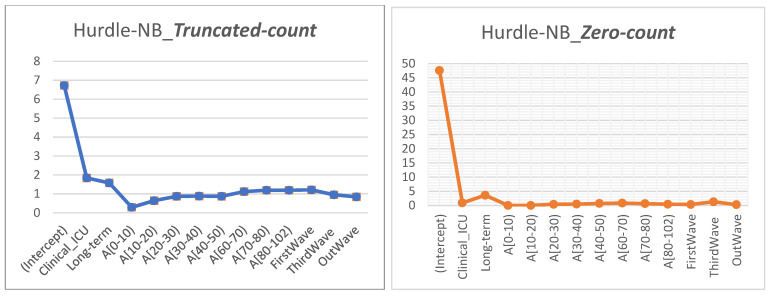
Visualization of IRR for the Poisson and NB models (**top** panel), and the Hurdle–NB truncated (**bottom left**) and zero-count effect plots (**bottom right**).

Interpretation (Hurdle–NB, positive count model or truncated Poisson) (see Table 6): Given the positive response (among those with positive counts), the average count was 6.72. The average LoS increased by 1.84-fold in the ICU setting compared with ordinary hospitalization and low-intensity stays among those with positive counts. The average LoS increased in the age groups of [60, 70), [70, 80), and 80+ by 1.12-, 1.19-, and 1.19-fold, respectively, and decreased by 0.29-, 0.64-, 0.88-, and 0.87-fold in the age groups of [0, 10), [10, 20), [30, 40), and [40, 50), respectively, whereas the age group of [20, 30) did not have a significant effect on the average LoS of those who had positive counts or a LoS greater than 0, all compared with the [50, 60) age group. The average LoS increased by 1.58-fold in long-term stays compared with regular hospitalization among those with positive counts. The average LoS increased by 1.21-fold during the first wave and decreased during the third wave and out waves by 0.95 and 0.84 times, respectively, for those with positive counts, compared with the second wave (chosen as the reference level). The exponentials of the coefficients or estimates for the Poisson model and NB model, and the Hurdle–NB model for count and zero Hurdle effect plots are shown in Figure 6.

### 3.3. Quantile Regression Models

**Figure 7:**
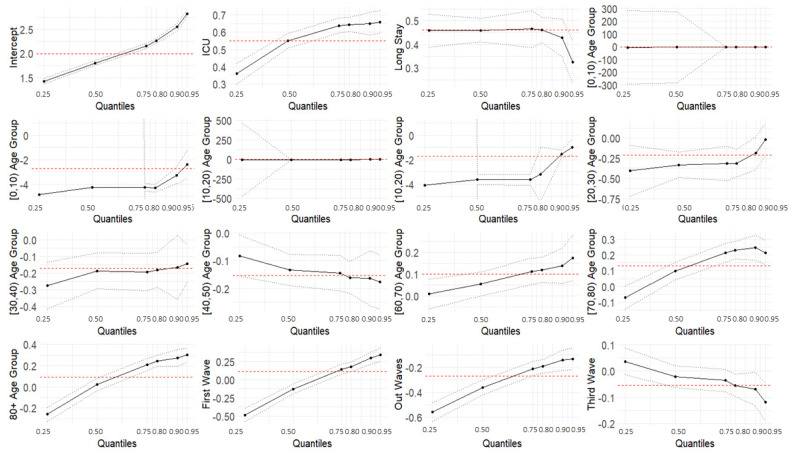
Quantile regression models.

The results of the quantile regression models are reported in Table 7. The intercept coefficients increased with the progression of the quantiles, ranging from 1.42 to 2.82. Covariates related to the ICU setting, hospitals with longer hospitalization, the first wave, and the out-waves were found to be statistically significant for all the modeled quantiles. Specifically, the coefficients for ICU settings, the first wave, and the out-wave were higher for higher quantile values, while coefficients for longer hospitalization and the third wave became lower as the quantile values increased. Differences in the third wave’s level coefficients were not statistically significant from the second wave’s values, except for the 0.8, 0.9, and 0.95 quantiles. Moreover, the coefficients were positive for ICU and longer hospitalization, while those for the out-wave were negative. The first wave’s coefficients were negative for the 0.25 and 0.5 quantiles, and they became positive for the higher quantile values. The age group covariate had a significant impact on the LoS. Estimated values for younger age groups ([0, 10) to [40, 50)) were found to be negative for all the quantiles, while most older groups presented positive estimates. The trend of coefficients for all the levels related to the age groups was to increase as the quantile values increased. A graphical depiction is presented in Figure 7.

## 4. Discussion

Researchers in the medical field are currently working to improve the quality and efficiency of health care systems and services in various ways, with LoS [26] being one of the efficiency and quality indicators. To the best of our knowledge, no previous study has examined the LoS in Bologna, Italy, among COVID-19 patients, and this is the first study to consider analyzing LoS using the best count fit model and comparing several models. This study also aimed to explore the hospital admission risk factors associated with LoS and presents a relatively novel method for modeling LoS using predictors.

The necessity of carefully picking a model that effectively describes the observed count data is demonstrated by the fact that the different statistical techniques produce mixed findings. First, we used the Poisson, negative binomial, Hurdle–Poisson, and Hurdle–NB regression models to model the effects of covariates on LoS. Second, we used quantile regression to model the impact of the variables on the quantile values of the response variable LoS. Compared with Poisson regression, the fit of the NB regression better tolerated the overdispersion in the data [20,24,25]. In addition to the overdispersion, the Hurdle models accounted for zero LoS more thoroughly [26]. The Hurdle–NB model was finally chosen on the basis of three criteria, including the AIC, the Vuong test, and Rootograms to understand the impacts of the predictors on the average LoS.

In our analysis, the median LoS was 6 days, which is comparable with the value reported in a similar study in Europe, USA, and the UK, but shorter than one in a study on China [12]. In this systematic review, the median hospital LoS ranged from 4 to 53 days within China (45 studies surveyed), and from 4 to 21 days outside China (eight studies surveyed). Similarly, a shorter LoS was also documented by the ISARIC (International Severe Acute Respiratory and Emerging Infections Consortium) report [40]. This report (which included data from 25 countries) described a median and IQR LoS of 4 and 1–9 days, respectively, which are substantially lower than in our study. Differences in LoS duration between nations can be explained by the different policies or strategies applied to control COVID-19 infection, or by the different population samples involved in the studies. Knowing the LoS or other adverse events in advance can help te health care systems organize the allocation of limited resources more efficiently.

Our results in Bologna are entirely consistent with those from studies which observed that older age (≥60 years) was associated with prolonged LoS [41,42,43]. Patients with ICU admissions had a longer LoS than those with only ordinary hospitalization, as these patients might need additional treatment or time when their disease reaches severe stages and they need more complex treatments. Staying in a long-term hospital was another contributing factor for a LoS longer than that in regular hospitals. This might be explained by the higher percentage of surgical operations and transfer rates, as well as restricted antibiotic use compared with other patients.

Usually, public hospitals manage acute patients’ LoS efficiently [44] in Italy. However, due to the new variants of the virus that causes COVID-19, the ability to assess or predict LoS will be more and more crucial in the future as a higher LoS is also associated with higher costs [45] and reduced capacity for other sanitary needs [46]. From this perspective, it is worth noting that we found that the LoS in Bologna was higher during the first wave—when the government proclaimed a state of emergency in response to an increase in the number of infection cases—and peaked around April 2021. However, during the third wave and out-wave, there was a drop, indicating that the chosen policies and strategies of the government and the health department, together with better clinical knowledge of the disease, have had a positive impact on the management of the patients.

We also used quantile regression to model the effects of the covariates on the quantile values of the response variable LoS, using a Poisson distribution, and to explore a range of conditional quantile functions, thereby exposing various forms of conditional heterogeneity and controlling for unobserved individual characteristics. The results from the quantile regression showed that the covariates related to the ICU setting, long-term hospitals, age groups, the first wave, and the out-waves were statistically significant for all the modeled quantiles. Specifically, for the ICU setting, the coefficients were higher for higher quantile values than ordinary hospitalization, meaning a longer LoS, and the effect was more pronounced for the higher quantiles. All the age groups, except for the [40, 50) group, showed an increasing trend with the quantiles. The wave also played a role: during the first wave, short LoSs were shorter (negative coefficients) and long LoSs were longer (positive coefficients) compared with those of the second wave. LoSs recorded in the out-wave period were shorter for all the quantiles (negative coefficients), and for the third wave, longer LoSs shortened (negative coefficients). The coefficients for long-term hospitalization became closer to zero as the quantile values increased, representing a less marked difference with respect to regular hospitalization.

The quantile regression models provided a more comprehensive overview of the effect of the covariates on a given phenomenon. As appears evident from this work, the same covariate can have a non-constant effect on different quantiles. This is valuable information for better modeling the LoS, which would not be controlled by simply evaluating the effect of the covariates on the mean values.

One limitation of our study is the absence of information on additional demographic and socio-economic variables, clinical characteristics, medical conditions, and laboratory tests for the patients, which might be among the most important factors that tend to increase the LoS. However, we have now started to analyze an extended, non-aggregated version of this dataset including all these variables.

COVID-19 cases are complex and still increasing worldwide, and it is difficult to predict when it will stop completely. Countries should plan and prepare for the worst-case scenarios, such as when different variants come into play (as in the case of Omicron at this time). In Italy, we have now entered into a fourth wave, so the wise use of limited health care resources is one of the most important priorities. Studies such as the present one might help health policymakers and managers to better plan the logistics of hospital settings, define priorities, and carry out a more accurate cost analysis. In addition, the information we have obtained and the method we used might serve as a tool or as a reference in the case of similar epidemics in the future.

## 5. Conclusions

Hurdle–NB had an excellent fit to the Bolognese COVID-19 data based on the AIC, the Vuong test, and Rootograms. On the basis of this model, we found a significant change in the average LoS with clinical setting, age group, type of hospital, and time in the pandemic (i.e., different waves). Moreover, these covariates, assessed via quantile regression models, showed a different impact on different quantiles and not only on the mean LoS values. The results obtained from our study can help to shed light on the risk factors that led to increased LoS among COVID-19 patients in Bologna, Italy. Hence, they could contribute to reducing unnecessary spending or arrangements, and help in identifying and managing prioritized settings for health professionals and policymakers, given the impact and the economic burden of the pandemic.

## Figures and Tables

**Table 1:** Descriptive statistics, frequency, and characteristics of the study sample (N = 13,203).

Predictors	N	%
Clinical setting	-	-
(Ordinary hospital) *	9818	74.36
(ICU, intensive + sub-intensive)	3385	25.64
Hospital-stay	-	-
Regular *	12,064	91.37
Long-term	1139	8.63
Age	-	-
[0–10)	412	3.12
[10–20)	181	1.37
[20–30)	167	1.26
[30–40)	401	3.04
[40–50)	1082	8.20
[50–60) *	1881	14.25
[60–70)	2237	16.94
[70–80)	2847	21.56
[80–102)	3991	30.23
Wave	-	-
First wave	2391	18.11
Second wave *	3369	25.52
Third wave	4173	31.61
Out-waves	3270	24.77

* Reference categories.

**Table 2:** Summary of estimates and standard errors (in brackets) of the Poisson and NB model regression coefficients.

	Poisson Model	NB Model
(Intercept)	2.00 ***	1.97 ***
	(0.01)	(0.03)
Clinical–ordinary	-	-
Clinical–ICU	0.55 ***	0.57 ***
	(0.01)	(0.02)
Regular	-	-
Long-term	0.46 ***	0.48 ***
	(0.01)	(0.03)
Age [0, 10)	−2.73 ***	−2.79 ***
	(0.08)	(0.09)
Age [10, 20)	−1.70 ***	−1.78 ***
	(0.07)	(0.10)
Age [20, 30)	−0.21 ***	−0.18 *
	(0.03)	(0.07)
Age [30, 40)	−0.17 ***	−0.15 **
	(0.02)	(0.05)
Age [40, 50)	−0.15***	−0.14 ***
	(0.01)	(0.03)
Age [50, 60)	-	-
Age [60, 70)	0.10 ***	0.09 ***
	(0.01)	(0.03)
Age [70, 80)	0.13 ***	0.14 ***
	(0.01)	(0.03)
Age [80, 102)	0.09 ***	0.11 ***
	(0.01)	(0.03)
First wave	0.11 ***	0.11 ***
	(0.01)	(0.02)
Second wave	-	-
Third wave	−0.05 ***	−0.04
	(0.01)	(0.02)
Out-wave	−0.27 ***	−0.26 ***
	(0.01)	(0.02)
AIC	131,254.90	82,120.2
BIC	131,359.73	82,232.52
Pseudo R^2^	0.77	0.23

*** *p* < 0.001; ** *p* < 0.01; * *p* < 0.05.

**Table 3:** Coefficients of the Hurdle–Poisson and Hurdle–NB models for the count process.

Hurdle–Poisson Model		Hurdle–NB Model
***(A) Truncated count process***		
	Estimate (Std. Error)	Estimate (Std. Error)
(Intercept)	2.01 *** (0.01)	1.91 *** (0.03)
Clinical–ordinary	-	-
Clinical–ICU	0.56 *** (0.01)	0.61 *** (0.02)
Regular	-	-
Long-term	0.42 *** (0.01)	0.46 *** (0.03)
Age [0, 10)	−0.98 *** (0.01)	−1.23 *** (0.15)
Age [10, 20)	−0.42 *** (0.07)	−0.45 ** (0.15)
Age [20, 30)	−0.16 *** (0.03)	−0.14 (0.08)
Age [30, 40)	−0.13 *** (0.02)	−0.13 * (0.05)
Age [40, 50)	−0.14 *** (0.01)	−0.14 *** (0.04)
Age [50, 60)	-	-
Age [60, 70)	0.11 *** (0.01)	0.11 *** (0.03)
Age [70, 80)	0.15 *** (0.01)	0.17 *** (0.03)
Age [80, 102)	0.13 *** (0.01)	0.17 *** (0.03)
First wave	0.17 *** (0.01)	0.19 *** (0.02)
Second wave	-	-
Third wave	−0.06 *** (0.01)	−0.05 * (0.02)
Out-wave	−0.19 *** (0.01)	−0.18 *** (0.02)
Log(theta)	-	0.436 *** (0.019)
***(B) Zero count process***		
	Estimate (Std.Error)	Estimate (Std. Error)
(Intercept)	3.86 *** (0.16)	3.86 *** (0.16)
Clinical–ordinary	-	-
Clinical–ICU	−0.11 (0.09)	−0.11 (0.09)
Regular	-	-
Long-term	1.29 *** (0.25)	1.29 *** (0.25)
Age [0, 10)	−4.62 *** (0.19)	−4.62 *** (0.19)
Age [10, 20)	−4.10 *** (0.22)	−4.10 *** (0.22)
Age [20, 30)	−0.93 ** (0.28)	−0.93 ** (0.28)
Age [30, 40)	−0.71 ** (0.23)	−0.71 ** (0.23)
Age [40, 50)	−0.35 (0.19)	−0.35 (0.19)
Age [50, 60)	-	-
Age [60, 70)	−0.16 (0.17)	−0.16 (0.17)
Age [70, 80)	−0.45 ** (0.15)	−0.45 ** (0.15)
Age [80, 102)	−0.83 *** (0.14)	−0.83 *** (0.14)
First wave	−1.08 *** (0.12)	−1.08 *** (0.12)
Second wave	-	-
Third wave	0.26 (0.14)	0.26 (0.14)
Out-wave	−1.20 *** (0.11)	−1.20 *** (0.11)

*** *p* < 0.001, ** *p* < 0.01, * *p* < 0.05.

**Table 4:** Model comparison using the Vuong test.

Model Comparison	Vuong Test Statistic	*p*-Value	Preferred Model
NB vs. Poisson	39.50	<0.0001	NB
Hurdle–Poisson vs. Poisson	21.20	<0.0001	Hurdle–Poisson
Hurdle–NB vs. Poisson	**39.81**	<0.0001	**Hurdle–NB**
Hurdle–Poisson vs. NB	−35.66	<0.0001	NB
Hurdle–NB vs. NB	10.17	<0.0001	Hurdle–NB
Hurdle–NB vs. Hurdle–Poisson	36.48	<0.0001	Hurdle–NB

Bold indicates the preferred model.

**Table 5:** Model comparison using AIC/BIC.

Model	AIC	BIC
Negative Binomial (NB)	82,120.2	82,232.5
**Hurdle–NB**	**81,353.9**	**81,571.1**
Poisson	131,254.9	131,359.7
Hurdle–Poisson	122,505.8	122,715.4

Bold indicates the preferred model.

**Table 6:** Estimates of coefficients: exponential (coef).

Hurdle-NB			NB
	**Count_Model**	**Zero_Hurdle_Model**	-
Intercept	6.72	47.57	7.20
Clinical–ICU	1.84	***0.90***	1.77
Long-term	1.58	3.61	1.61
Age [0, 10)	0.29	0.01	0.06
Age [10, 20)	0.64	0.02	0.17
Age [20, 30)	***0.87***	0.40	0.84
Age [30, 40)	0.88	0.49	0.86
Age [40, 50)	0.87	***0.71***	0.87
Age [60, 70)	1.12	***0.85***	1.10
Age [70, 80)	1.19	0.64	1.15
Age [80, 102)	1.19	0.44	1.12
First wave	1.21	0.34	1.11
Third wave	0.95	***1.30***	***0.96***
Out-wave	0.84	0.30	0.77

Bold italic font indicates the non-significant variables.

**Table 7:** Quantile regression models for count data.

Quantile Regression Models						
	Estimate (Std. Error)					
	0.25	0.5	0.75	0.8	0.9	0.95
(Intercept)	1.42 (0.03) ***	1.80 (0.02) ***	2–16 (0.03) ***	2.25 (0.02) ***	2.55 (0.04) ***	2.82 (0.03) ***
Clinical–Ordinary						
Clinical–ICU	0.36 (0.03) ***	0.55 (0.02) ***	0.64 (0.02) ***	0.64 (0.02) ***	0.65 (0.03) ***	0.66 (0.03) ***
Regular						
Long-term	0.46 (0.03) ***	0.46 (0.02) ***	0.46 (0.04) ***	0.46 (0.03) ***	0.43 (0.04) ***	0.32 (0.05) **
Age [0, 10)	−4.84 (147.26)	−4.27 (142.09)	−4.26 (0.016) ***	−4.32 (0.15) ***	−3.27 (0.34) ***	−2.38 (0.57) **
Age [10, 20)	−4.08 (239.49)	−3.60 (0.21) ***	−3.64 (0.22) ***	−3.19 (1.12) **	−1.52 (0.16) ***	−0.98 (0.30) *
Age [20, 30)	−0.40 (0.16)	−0.33 (0.08) **	−0.31 (0.11) **	−0.31 (0.09) **	−0.19 (0.10)	−0.02 (0.10)
Age [30, 40)	−0.27 (0.07) *	−0.18 (0.05) **	−0.19 (0.06) **	−0.18 (0.05) **	−0.16 (0.10)	−0.14 (0.06)
Age [40, 50)	−0.08 (0.04)	−0.13 (0.03) **	−0.14 (0.03) **	−0.16 (0.03) **	−0.16 (0.05) *	−0.18 (0.05) **
Age [50, 60)						
Age [60, 70)	0.009 (0.03)	0.05 (0.03)	0.11 (0.03) **	0.11 (0.02)	0.14 (0.03) **	0.17 (0.05) *
Age [70, 80)	−0.07 (0.04)	0.10 (0.03) **	0.21 (0.03) **	0.22 (0.03) **	0.24 (0.03) **	0.21 (0.04) **
Age [80, 102)	−0.26 (0.03) **	0.02 (0.03)	0.21 (0.03) **	0.24 (0.03) ***	0.27 (0.04) **	0.30 (0.03) ***
First wave	−0.48 (0.04) ***	−0.13 (0.03) **	0.14 (0.03) **	0.17 (0.03) **	0.29 (0.04) **	0.34 (0.05) **
Second wave						
Third wave	0.04 (0.03)	−0.02 (0.02)	−0.03 (0.02)	−0.05 (0.02)	−0.07 (0.03)	−0.12 (0.04) *
Out-wave	−0.57 (0.04) ***	−0.36 (0.03) ***	−0.21 (0.03) **	−0.19 (0.03) **	−0.14 (0.04) **	−0.13 (0.04) *

*p* < 0.05, * *p* < 0.01, ** *p* < 0.001, *** *p* = 0.0001.

## Data Availability

The data are not publicly available due to ethical restrictions.
